# An Attempt at a Molecular Prediction of Metastasis in Patients with Primary Cutaneous Melanoma

**DOI:** 10.1371/journal.pone.0049865

**Published:** 2012-11-14

**Authors:** Melanie Gschaider, Friederike Neumann, Bettina Peters, Florian Lenz, Michael Cibena, Malgorzata Goiser, Ingrid Wolf, Jörg Wenzel, Cornelia Mauch, Wolfgang Schreiner, Stephan N. Wagner

**Affiliations:** 1 Division of Immunology, Allergy and Infectious Diseases, Department of Dermatology, Medical University of Vienna, Vienna, Austria; 2 Section for Biosimulation and Bioinformatics, Center for Medical Statistics, Informatics and Intelligent Systems, Medical University of Vienna, Vienna, Austria; 3 Project Management Agency, Member of German Aerospace Centre, Health Research, Bonn, Germany; 4 Department of Dermatology, Medical University of Graz, Graz, Austria; 5 Department of Dermatology, University of Bonn, Bonn, Germany; 6 Department of Dermatology, University of Cologne, Cologne, Germany; The Moffitt Cancer Center & Research Institute, United States of America

## Abstract

**Background:**

Current prognostic clinical and morphological parameters are insufficient to accurately predict metastasis in individual melanoma patients. Several studies have described gene expression signatures to predict survival or metastasis of primary melanoma patients, however the reproducibility among these studies is disappointingly low.

**Methodology/Principal Findings:**

We followed extended REMARK/Gould Rothberg criteria to identify gene sets predictive for metastasis in patients with primary cutaneous melanoma. For class comparison, gene expression data from 116 patients with clinical stage I/II (no metastasis) and 72 with III/IV primary melanoma (with metastasis) at time of first diagnosis were used. Significance analysis of microarrays identified the top 50 differentially expressed genes. In an independent data set from a second cohort of 28 primary melanoma patients, these genes were analyzed by multivariate Cox regression analysis and leave-one-out cross validation for association with development of metastatic disease. In a multivariate Cox regression analysis, expression of the genes Ena/vasodilator-stimulated phosphoprotein-like (EVL) and CD24 antigen gave the best predictive value (p = 0.001; p = 0.017, respectively). A multivariate Cox proportional hazards model revealed these genes as a potential independent predictor, which may possibly add (both p = 0.01) to the predictive value of the most important morphological indicator, Breslow depth.

**Conclusion/Significance:**

Combination of molecular with morphological information may potentially enable an improved prediction of metastasis in primary melanoma patients. A strength of the gene expression set is the small number of genes, which should allow easy reevaluation in independent data sets and adequately designed clinical trials.

## Introduction

Human melanoma is the most malignant skin cancer [1] and its incidence is still increasing in most developed countries. According to the WHO- World Cancer Report 2008, melanoma is the fifth most common cancer in males and the sixth in females in North America. In Europe, melanoma is the eighth and the sixth most common cancer in males and in females, respectively [2]. Notably, melanoma is the most common skin cancer in Caucasian females aged 25–29 [3]. Proclivity for metastasis and therapeutic resistance are hallmarks of melanoma. After metastatic spread to vital organs, the average life span of patients is less than a year [4]. Despite the recent developments with novel targeted therapies, the key to improved survival remains early detection and surgery of primary melanoma.

Although numerous molecular events have been associated with development and progression of melanoma [1], the American Joint Committee on Cancer (AJCC) Melanoma Staging and Classification is still the most important system for disease classification [5]. This system allows stratification of individual patients into patient cohorts with comparable disease outcome, mainly on the basis of a TNM-based tumor staging. In patients with primary cutaneous melanoma (clinical stages I and II disease), the most useful prognostic indicators to date remain morphological features such as Breslow depth, the presence or absence of ulceration and the mitotic rate (MR; mitoses per mm^2^) [5]–[7]. However, on basis of these criteria it is not possible to provide patients with accurate individual prognostic information at the time of diagnosis [8]. This can be exemplified by the biological behavior of “thick” and “thin” primary melanomas: although thick lesions have a much higher risk for metastasis than do their thinner counterparts, there are also thin cutaneous melanomas that metastasize early [9]. The consequences of the lack of valuable individualized prognostic information are immense. As state-of-the-art procedure, clinical stage II melanoma patients are frequently included into adjuvant treatment trials [8]. However, as only around 50% of these patients will develop metastatic disease later on [10], several thousands of melanoma patients are continuously over-treated. In addition, the unnecessary treatment of half of these patients has also significant negative implications on trial design, required patient numbers and, as a result, on drug development.

In the last years several attempts have been made to develop individualized prediction of metastasis from a merely morphology-based into a state-of-the-art molecular approach. Within the past decade several gene expression studies have reported molecular predictors for disease outcome in melanoma, may it be survival or development of metastasis, with disappointingly low congruence [11]–[24]. As a result, gene expression signatures have neither been established as molecular predictors of metastasis and overall survival nor changed clinical practice so far and several recommendations for analysis and reporting of microarray data studies have been developed [25]–[28].

In this study, we followed the guidelines on statistical analysis and reporting of gene expression data for cancer outcome [26] and the REporting recommendations for tumor MARKer prognostic studies (REMARK/Gould Rothberg criteria) as adapted for gene expression microarray studies [27]–[30]. We have generated two independent gene expression datasets from two independent patient cohorts with primary cutaneous melanomas. The one dataset was used for class comparison between non-metastatic and metastatic primary melanomas by serial analyses of gene expression (SAGE™) and the other dataset for class prediction, *i.e.* metastasis during clinical follow up. The Cox proportional hazards model followed by leave-one-out cross validation (LOOCV) revealed two genes whose expression was associated best with metastasis, namely the genes encoding the Ena/vasodilator-stimulated phosphoprotein-like (EVL) and CD24 antigen (CD24).

## Results

### Class comparison identifies genes differentially expressed in human non-metastatic and metastatic primary cutaneous melanoma samples

To identify the gene expression profiles of individual melanoma patients, the cDNA from each tumor was hybridized against a common skin reference as described [31], [32]. Significance analysis of microarrays (SAM) was performed to compare gene expression profiles between 116 non-metastasized (clinical stage I/II) and 72 metastasized (clinical stage III/IV) primary melanomas. The top 50 differentially expressed genes (p<0.01) were used for further analysis (Table S1).

### Cox-regression analysis identifies genes associated with metastasis in an independent patient cohort

The top 50 genes identified by class comparison were used in the second independent gene expression dataset of 28 primary melanomas for prediction of metastasis.

Affymetrix probe sets matching these 50 genes were determined using the GeneAnnot-database [33]. Eighty-six corresponding probe sets could be identified matching 43 different genes (Table S1). When classified into Gene Ontology (GO)-clusters (National Institute of Allergy and Infectious Diseases Database for Annotation, Visualization and Integrated Discovery (DAVID) Bioinformatics Resources 6.7; http://david.abcc.ncifcrf.gov), most of these genes were assigned to immune defense response and regulation of cell proliferation.

**Figure 1:**
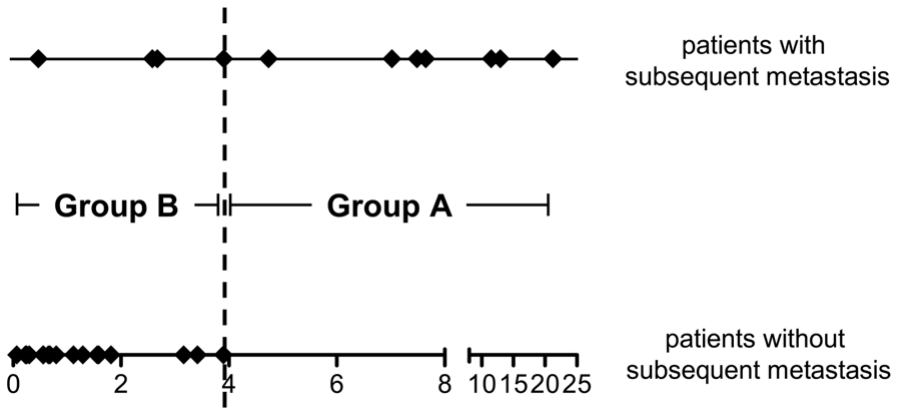
Prediction of metastasis by the EVL and CD24 gene set. Patients without (lower horizontal line) and with (upper horizontal line) subsequent metastasis ranked according to the risk scores (x-axis) as defined by Cox regression are plotted as diamonds. The black vertical line indicates the cutoff position maximizing precise prediction of metastasis (between groups A and B).

These 43 genes were used as independent variables in a multivariate Cox regression analysis. The two probe sets with the best predictive value comprised the Affymetrix probe set IDs 217838_s_at (HR = 0.288, p = 0.001) and 208651_x_at (HR = 2.034, p = 0.016) matching the genes encoding Ena/vasodilator-stimulated phosphoprotein-like (EVL) and CD24 antigen (CD24), respectively (Table 1). The expression of these two genes allowed the separation of primary melanoma samples into two groups based on the risk score calculated by Cox regression as shown in Figure 1. Here, the patients' ranking according to the risk scores distributed primary melanoma patients without or with subsequent metastasis along a scale with the former patients at the lower line and the latter patients at the upper line. When the cutoff for group assignment was set as the average of the risk scores of two adjacent patients maximizing precise prediction of metastasis, one group (group A) comprised 8 of the 11 patients with subsequent metastasis and the other group (group B) 20 patients who included all of the 17 patients without subsequent metastasis. Patients with metastasis showed a lower expression of EVL and a higher expression of CD24 as compared to patients without metastasis.

**Table 1:** Predictive gene expression set of the Cox proportional hazards model as obtained in a forward stepwise regression procedure.

Gene	Parameter estimate*	Standard error	p-value	HR (95% CI)
217838_s_at (EVL)	−2.492	0.762	0.001	0.288 (0.136–0.607)
208651_x_at (CD24)	1.420	0.592	0.016	2.034 (1.139–3.632)

* A shrinkage factor of 0.74 should be applied on the parameter estimates to prevent an overestimation due to the limited number of patients [59].

Leave-one-out cross-validation analysis was used on the 28 patient dataset to evaluate the model-based gene set for hypothetical prediction of metastasis for each patient. Here, one sample was withdrawn from the initial 28-sample dataset, leaving a temporary 27-sample training set and one left out-sample. On the training set, the gene set obtained from Cox regression is then used to classify the previously left out-test sample [26]. Performing LOOCV with the EVL and CD24 gene set with the cut off described above, a specificity of 88.2% (95% CI = 63.6–98.5%) and a sensitivity of 45.5% (95% CI = 16.8–76.2%) were estimated. Note that the range of confidence intervals (CIs) reflects the rather limited sample number.

### Metastasis prediction in human primary cutaneous melanomas

We next asked, whether gene expression could possibly add to the predictive power of the most important morphological parameter, Breslow depth. In a multivariate Cox proportional hazards model we evaluated the combination of Breslow depth either with ulceration, mitotic rate or gene expression for additional effects on prediction of metastasis.

**Figure 2:**
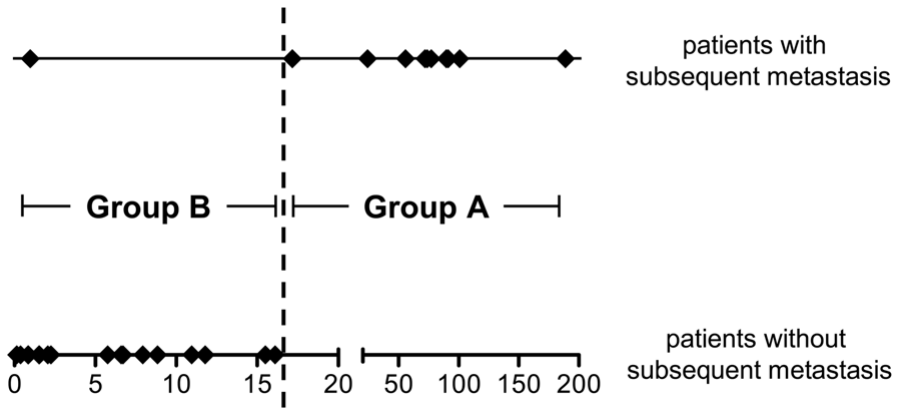
Prediction of metastasis by Breslow depth plus the EVL and CD24 gene set. Patients without (lower horizontal line) and with (upper horizontal line) subsequent metastasis, again plotted as diamonds and ranked according to the risk scores (x-axis) as defined by Cox regression. The black vertical line indicates the cutoff position maximizing precise prediction of metastasis (between groups A and B).

While addition of ulceration or mitotic rate did not improve the predictive value of Breslow depth (Table 2), addition of gene expression to Breslow depth did (EVL (HR = 0.345, p = 0.01); CD24 (HR = 2.686, p = 0.012), Table 2). The area under the curve (AUC) by Breslow depth alone was 80.2%, by gene expression alone 89.8% and increased to 93.0% by the combination of both. When patients were ranked according to risk scores calculated by Cox regression and the cutoff for group assignment was set as to maximize precise prediction of metastasis, the combination of Breslow depth with gene expression allowed precise prediction in 10 of the 11 primary melanoma patients with subsequent metastasis (group A, Figure 2).

**Table 2:** Hazard ratios and confidence intervals for Breslow depth in combination with morphological parameters, or together with the gene expression set as obtained by a multivariate Cox proportional hazards model.

Prognostic factor	Parameter estimate	Standard error	p-value	HR** (95% CI)
Breslow depth and MR*	0.3600.010	0.1800.024	0.0500.681	1.197 (1.003–1.428)1.051 (0.828–1.334)
Breslow depth and ulceration	0.3500.453	0.1480.676	0.0190.503	1.189 (1.029–1.375)1.573 (0.418–5.912)
Breslow depth and EVL and CD24	0.391−2.1281.976	0.1920.8270.786	0.0420.0100.012	1.216 (1.007–1.468)0.345 (0.153–0.776)2.686 (1.243–5.804)

* MR, mitotic rate, calculation with n = 22 samples, due to missing reports for MR;

** HR, hazard ratio referring to a change of 0.5 units in gene expression and Breslow depth, of 1 unit in ulceration and of 5 units in MR.

Consistent with the results obtained by the Cox proportional hazards model, there was a low correlation between Breslow depth and the model risk score calculated from the two genes (correlation coefficient = 0.338, p = 0.041), suggesting the latter as a potential independent predictor for metastasis. In contrast, the morphological parameter mitotic rate correlated significantly with Breslow depth (correlation coefficient = 0.578, p = 0.005).

## Discussion

Although genomic information has significantly contributed to the understanding of the cell biology and also to the therapy of melanoma, molecular information has failed so far to provide robust information for individualized prediction of the clinical course of patients. This is best exemplified by studies using gene expression microarrays for class prediction such as survival or metastasis. For example, in 7 independent and prominently published studies on class prediction for survival in human melanoma biopsies a total number of around 590 different candidate genes (either alone or as part of gene expression signatures) were proposed as molecular markers [11], [13]–[15], [22]–[24]. However, only one single gene, *i.e.* lymphotoxin beta, was identified by at least three of these studies [11], [14], [15]. The same heterogeneity can be observed in gene expression studies on class prediction for metastasis. In 7 independent studies, a total number of around 280 different candidate genes, either alone or as part of a gene expression signature, were presented as molecular markers [12], [16]–[21]. Only 9 of these genes were shared by at least two of these studies [16]–[21]. In immunohistochemical or gene expression profiling studies on the prognostic value of preselected markers or marker combinations, increased expression of osteopontin (SPP1) has been described to predict relapse-free and disease-specific survival, respectively [23], [34]. Interestingly, we identified osteopontin as one of the top 50 differentially expressed genes in class comparison, however, osteopontin did not show up in the two probe-set with the best predictive value in the class prediction dataset.

In 2007, Dupuy and Simon critically reviewed a number of 90 published microarray studies on cancer outcome and described several issues heavily compromising the validity and the reproducibility of these studies (Dupuy and Simon, 2007). In view of the many pervasive mistakes and misunderstandings in studies published even in high impact journals, the REMARK/Gould Rothberg criteria were developed for statistical analysis and reporting of microarray studies for clinical outcomes of patients with primary cutaneous melanoma [29], [30]. Adherence to these guidelines is viewed as critical not only in translational studies in melanoma [27], [28], but also in other cancers [35]. By using a cohort study design, applying a multivariate proportional hazards analysis, including a detailed description of methods, providing details of positive and negative controls, and reporting the data with hazard ratios and 95% confidence intervals, we evaluated primary cutaneous melanomas closely following these recommendations. Most importantly, we strictly separated the datasets used for identification of outcome-related genes and the ones used for supervised classification to avoid preliminary usage of the test samples used for supervised prediction.

Interestingly, the use of a separate test set for supervised prediction appears to be a gold standard to some authors, but in many studies the use of a separate test set almost invariably brought more confusion than clarity. Instead, the gold standard should rather be a proper validation of the classifier performance and this can be achieved through a cross-validation procedure as well [26]. This was the reason why we did not use a dual validation approach with a so-called test set. Furthermore, we performed a LOOCV to report the fully specified gene set with its parameters and calculations for sensitivity and specificity. The latter is particularly important in a clinical setting where sensitivity and specificity are more relevant than global accuracy [26]. Finally, we are presenting the performance in the way the data have been trained, namely by comparing the true and the predictive disease outcome (*i.e.* metastasis).

The identified gene sequences encode CD24 and EVL and both have documented established and/or proposed biological functions relevant to cancer cell biology. CD24 is a GPI-anchored mucin-like membrane protein originally identified as a signal-transducing molecule on the surfaces of most human B cells [36] and a ligand for the cell adhesion molecule P-selectin [37]. CD24 is rarely expressed on normal cells, but may be highly expressed on stem cells of cancers including pancreatic, ovarian and colorectal ones (reviewed in [38]). Up-regulation of CD24 expression has documented negative prognostic impact in patients with cancers as diverse as ovarian, breast, prostate, hepatocellular, non-small cell lung, colorectal cancer and gastric adenocarcinoma (reviewed in [39]). In melanoma, CD24 is part of the CD44+CD133+CD24+ stem cell-like immunophenotype in B16-F10 mouse melanoma cells [40] and is upregulated in these cells during in vivo tumor formation [41]. In humans, primary melanomas have been shown to express CD24 [42] which, together with our data, is consistent with the increasing evidence that solid cancers including melanomas can acquire early in their evolution genomic alterations predicting significant metastatic potential [43].

EVL (Ena/VASP-like) is a member of the Ena/VASP (Enabled/vasodilator-stimulated phosphoprotein) family of proteins, which is a key regulator of cytoplasmic actin at sites of actin remodeling such as focal adhesions, the tips of filopodia or cell-matrix and cell-cell junctions [44]. While Ena/VASP proteins can be upregulated in some human cancers and their expression may be increasing with progression of the disease [45], [46], the exact functional consequences of expression changes of Ena/VASP proteins to cancer cell biology remain elusive. The tumor-promoting function of this protein family can be significantly modulated by expression levels not only in tumor cells but also in the surrounding tumor environment as exemplified for melanoma in the B16 allograft model, where growth of VASP-expressing tumor cells was largely impaired in VASP-deficient animals [47]. Furthermore, the mutual functional compensation of family members [48], the modulation of activity by expression and intracellular distribution of their respective ligands [49], [50] as well as by signaling pathways such as EGF-R signaling [51] and additional functions such as the recently described involvement of EVL in homologous recombinational repair of double-strand DNA breaks [52], may all have significant and sometimes opposite impact on tumor cell development and progression.

Taken together our data show that, in contrast to morphological parameters such as mitotic rate and ulceration, gene expression analysis may potentially add to the predictive value of the most important indicator of primary cutaneous melanoma, Breslow depth. Whether this information is becoming translationally relevant is subject to further evaluation in independent datasets and adequately designed clinical trials.

## Materials and Methods

### Class comparison dataset

#### Ethics Statement

This study was approved by the Local Ethic Committees at the Universities of Cologne, Bonn and Aachen and has fulfilled the Declaration of Helsinki Principles for human research. All patients signed a consent form to participate in this study.

#### DNA Collection and RNA Preparation

Primary melanoma tissue samples were either collected at the Departments of Dermatology at the Universities of Cologne, Bonn or Aachen. Each department performed its histological and immunohistochemical routine procedures. Reference histology for melanoma biopsies was done at the Department of Dermatology of the University of Cologne. Healthy skin control samples were obtained at the Department of Dermatology at the Universities of Bonn or Cologne and re-examined at the Department of Dermatology at the University of Bonn. Primary melanoma lesions were classified at the time of surgery based on a combination of clinicopathological features and the AJCC 2002 staging system [10], [53]. 116 samples of non-metastasized (clinical stage I/II) primary melanomas and 72 samples of metastasized (clinical stage III/IV) primary melanomas were collected that way.

Immediately after surgery, skin biopsies were flash-frozen in liquid nitrogen. Total RNA from skin excision biopsies was isolated as described earlier [31]. Using the TriReagent (Sigma, St. Louis, MO) and the Nucleo-Spin 96 RNA Kit (Macherey & Nagel, Dueren, Germany), quantification of RNA was performed by photometrical measurements on a 2.100 Bioanalyzer (Agilent Technologies, Palo Alto, CA).

#### Gene expression analysis by SAGE™ and PIQOR™

Serial analyses of the gene expression (SAGE™) analysis of total RNA was performed as previously described [31] according to the “MicroSAGE Detailed Protocol”, available at http://www.sagenet.org, with minor modifications. Each SAGE library was obtained from a pool of mRNAs derived from 20 to 22 biopsies of melanomas and normal skin, respectively, to minimize biopsy related variations [31]. A topic-defined PIQOR™ (Parallel Identification and Quantification of RNAs) microarray (Miltenyi Biotec GmbH, Bergisch Gladbach, Germany) was designed on the basis of SAGE™ analysis according to the procedures previously described [31]. This microarray was used to obtain a gene expression profile for each individual melanoma sample. Here, Cy5–labeled RNA from tumor samples was hybridized against a Cy3–labeled common skin reference pool as described [31], [32]. Hybridization, scanning, and data analysis were performed according to the PIQOR™ protocol [31], [54], [55] and in compliance with the MIAME (Minimum information about a microarray experiment) standards.

### Statistical analysis

The formula by Audic and Claviere [56] was applied to gene expression levels obtained by SAGE™ analysis to identify genes differentially expressed by non-metastatic and metastatic primary melanoma versus healthy skin samples (p<0.01) [31], [55]. The expression levels of these genes were subsequently analyzed in PIQOR™-derived data by SAM using the standardized Wilcoxon rank test to identify the top 50 genes differentially expressed between individual non-metastatic and metastatic primary melanoma samples (p<0.01) [32]. The SPSS™ software (version 14) was used for computer-based statistical analyses.

### Class prediction dataset

#### Ethics Statement

All research involving human participants was approved by the institutional review board at the University of Essen and granted an exemption. The study has fulfilled the Declaration of Helsinki Principles. All patients gave written informed consent.

#### RNA preparation and data collection

Fresh primary cutaneous melanoma biopsies from twenty-eight patients were processed as described [21]. The samples were collected from 1992 to 2001 and annotated with clinical information including follow up with a median observation period of 35 months (14 and 72, respectively, for patients with and without metastasis), ranging from 1–147 months. Two histopathologists diagnosed each tumor specimen independently. The clinical and histopathological characteristics of the patients in the class prediction dataset are summarized in Table 3.

**Table 3:** Clinical and histopathological characteristics of the class prediction dataset.

Characteristics	
Age at time of resection, years (median, range)	59.6 (29.4–85.1)
Clinical follow up, months (median, range)	35 (1–147)
Gender, no.	
Female	11
Male	17
Breslow depth, mm (median, range)	1.81 (0.35–7.50)
Ulceration, no.	
Absent	20
Present	8
MR*, no. per mm^2^ (median, range)	2.5 (0–45)
Subtype, no.	
Superficial spreading	28
Body site, no.	
Extremities	13
Trunk	9
Head/Neck	6

* n = 22, due to missing reports.

Cellular RNA was extracted by guanidinium thiocyanate and cesium chloride centrifugation. The purification from the remaining melanin was performed using the Qiagen RNeasy Fibrous Tissue Mini Kit. The preparation of cRNA was carried out according to the GeneChip Expression Analysis Technical Manual (Affymetrix), and hybridized onto HU133A chips (Affymetrix). A GeneArray@ 2500 Scanner (Affymetrix) was used for scanning and the quality of raw microarray profiles assessed as described [21]. The data are accessible through GEO Series accession no. GSE8401.

#### Statistical analysis

Microarray probe intensity data from 28 primary melanoma patients were read from Affymetrix CEL files and processed with the Genespring GX11.5 software (Agilent Technologies Inc., Santa Clara, CA). The Robust Multi-Array Average algorithm (RMA) [57] was used to calculate expression measures from raw data. The expression measures of the 86 probe sets covering the top differentially expressed genes as identified in the independent class comparison set were exported as spreadsheet to the SAS System V9.2 (SAS Institute Inc., Cary, NC) for further statistical analysis.

The Cox proportional hazard model [58] was used with the pre-specified endpoint (documented metastasis), the time from first diagnosis to documented metastasis and censored time values (time from first diagnosis to last clinical visit) for patients without metastasis. The PHREG procedure of the SAS System V9.2 (SAS Institute Inc., Cary, NC) was used for statistical computations. All 86 RMA-normalized probe sets were subjected to a forward stepwise Cox regression. Significance levels for entering and removing effects were set to p = 0.05. Individual risk scores were defined as a linear combination of the patient's gene expression values multiplied by the parameter estimates (coefficients) of the two model effects (probe sets) specified by Cox regression modeling. The hazard ratios of the model effects compare the hazard for a change of 0.5 units in the respective effect.

In a LOOCV we tried to evaluate the predictive capability of the model-based gene set. The outcome (*i.e.* metastasis) of a particular patient was predicted from the patient's risk score as calculated from the Cox model fitted to all other patients. The predicted outcome was compared to the real outcome to estimate the error rate (*i.e.* the probability of incorrectly classifying a future case) of the predictor on the basis of a suitably chosen decision rule (“cutoff”). The cross-validated risk scores were used as the covariate in another (univariate) Cox regression model. The resulting parameter estimate was used as a shrinkage factor for the -due to the limited number of patients and the forward modeling procedure- expectedly overestimated coefficients of the original Cox model.

The Pearson Correlation was used to compute the correlation between Breslow depth, MR or the model risk score.

The area under the curve (AUC) was calculated by summarizing the relative frequencies of true positive predictions over the relative frequencies of false positive predictions from Cox regression (risk scores), *i.e.* a “receiver operating characteristic” (ROC) analysis using the binary outcome of documented metastasis or not.

## Supporting Information

Table S1 Top 50 genes differentially expressed between human non-metastatic and metastatic primary melanomas of the class comparison data set.
(DOCX)
