# Supplementary material for: An Attempt at a Molecular Prediction of Metastasis in Patients with Primary Cutaneous Melanoma
Source: PLoS One. 2012 Nov 14;7(11):e49865. doi: 10.1371/journal.pone.0049865 (PMC3498185; doi:10.1371/journal.pone.0049865)
Supplement: Table S1 — Top 50 genes differentially expressed between human non-metastatic and metastatic primary melanomas of the class comparison data set. (DOCX) [file pone.0049865.s001.docx]

**Supporting Information**

**Table S1.** Top 50 genes differentially expressed between human non-metastatic and metastatic primary melanomas

| **Gene*** | **Affymetrix probe set ID** | **Entrez Gene ID** | **Description** |
| --- | --- | --- | --- |
| IL1B | 205067_at, 39402_at | 3553 | interleukin 1, beta |
| PCNA | 201202_at | 5111 | proliferating cell nuclear antigen |
| CD24 | 208650_s_at, 208651_x_at, 209771_x_at, 209772_s_at, 216379_x_at, 266_s_at | 100133941 | CD24 molecule |
| DARC | 208335_s_at | 2532 | Duffy blood group, chemokine receptor |
| CXCL1 | 204470_at | 2919 | melanoma growth stimulating activity, alpha |
| MGP | 202291_s_at | 4256 | matrix Gla protein |
| MMP3 | 205828_at | 4314 | matrix metallopeptidase 3 |
| SPP1 | 209875_s_at, 218058_at, 221268_s_at, 48580_at | 6696 | secreted phosphoprotein 1 |
| *GJB6* |  | 10804 | gap junction protein, beta 6, 30kDa |
| BMP4 | 211518_s_at, 201405_s_at, 213504_at | 652 | bone morphogenetic protein 4 |
| BAG5 | 202984_s_at, 202985_s_at | 9529 | BCL2-associated athanogene 5 |
| HSP90AB1 | 200064_at, 214359_s_at | 3326 | heat shock 90kD protein 1, beta |
| CCL19 | 210072_at | 6363 | chemokine (C-C motif) ligand 19 |
| CXCL14 | 218002_s_at | 9547 | chemokine (C-X-C motif) ligand 14 |
| APOC2 | 204561_x_at | 344 | apolipoprotein C-II |
| PTGDS | 211663_x_at, 211748_x_at, 212187_x_at | 5730 | prostaglandin D2 synthase 21kDa (brain) |
| SOD2 | 215078_at, 215223_s_at, 216841_s_at, 221477_s_at | *6648* | superoxide dismutase 2, mitochondrial |
| LCN2 | 212531_at | 3934 | lipocalin 2 |
| CALR | 200935_at, 212953_x_at, 214315_x_at | 811 | Calreticulin |
| CCT4 | 200877_at | 10575 | chaperonin containing t-complex polypeptide 1, delta subunit |
| PDIA6 | 207668_x_at, 208638_at, 208639_x_at, 216640_s_at | 10130 | protein disulfide isomerase family A, member 6 |
| S100A12 | 205863_at | 6283 | S100 calcium binding protein A12 |
| CBX3 | 200037_s_at, 201091_s_at | 11335 | chromobox homolog 3 |
| LDHB | 201030_x_at, 213564_x_at | 3945 | lactate dehydrogenase B |
| CORO1C | 221676_s_at | 23603 | coronin, actin binding protein, 1C |
| CYP27B1 | 205676_at | 1594 | cytochrome P450, family 27, subfamily B, polypeptide 1 |
| CORO1A | 209083_at | 11151 | coronin, actin binding protein, 1A |
| CRIP1 | 205081_at | 1396 | cysteine-rich protein 1 (intestinal) |
| IGFBP7 | 201162_at, 201163_s_at | 3490 | insulin-like growth factor binding protein 7 |
| CALML5 | 220414_at | 51806 | calmodulin-like 5 |
| *PLAC9* |  | 219348 | placenta-specific 9 |
| MDH1 | 200978_at | 4190 | soluble malate dehydrogenase |
| SERPINB13 | 211361_s_at, 211362_s_at, 217272_s_at, 216257_at, 216258_s_at | 5275 | serpin peptidase inhibitor, clade B (ovalbumin), member 13 |
| ISG15 | 205483_s_at | 9636 | ISG15 ubiquitin-like modifier |
| MX1 | 202086_at | 4599 | Myxoma resistance protein 1 |
| *CCDC80* |  | 151887 | coiled-coil domain containing 80 |
| TNA | 205200_at | 7123 | Tetranectin |
| *ZBTB4* |  | 57659 | zinc finger and BTB domain containing 4 |
| FOLR2 | 204829_s_at | 2350 | folate receptor 2 (fetal) |
| KDELR2 | 200698_at, 200699_at, 200700_s_at | 11014 | KDEL endoplasmic reticulum protein retention receptor 2 |
| ADN | 205382_s_at | 1675 | Adipsin |
| MFAP4 | 212713_at | 4239 | microfibrillar-associated protein 4 |
| TRAC | 207760_s_at, 209670_at, 209671_x_at, 210972_x_at, 211902_x_at, 215524_x_at, 208888_s_at, 208889_s_at, 211667_x_at, 215205_x_at, 217063_x_at | 28755 | T cell receptor alpha constant |
| TRBC | 210915_x_at, 213193_x_at | 28639 | T cell receptor beta constant 1 |
| EVL | 217838_s_at | 51466 | Enah/Vasp-like |
| FCGBP | 203240_at | 8857 | Fc fragment of IgG binding protein |
| *KAP10* |  | 10870 | hematopoietic cell signal transducer |
| RPS27L | 218007_s_at | 51065 | ribosomal protein S27-like |
| *S100A7L1* |  | 338324 | S100 calcium binding protein A7A |
| *LCE1B* |  | 353132 | late cornified envelope 1B |

^*^ For genes given in italics no corresponding Affymetrix probe set ID could be identified.
